# Supplementary material for: HIVprotI: an integrated web based platform for prediction and design of HIV proteins inhibitors
Source: J Cheminform. 2018 Mar 9;10:12. doi: 10.1186/s13321-018-0266-y (PMC5845081; doi:10.1186/s13321-018-0266-y)
Supplement: Supplementary file 2 — Additional file 2. Source code of HIVProtI web server. [file 13321_2018_266_MOESM2_ESM.zip › HIVprotI_Source-code/hivprotI/weka-3-6-6/doc/weka/gui/visualize/VisualizePanelEvent.html]

VisualizePanelEvent


---


|  |  |  |
| --- | --- | --- |
| **Overview** \| **Package** \| **Class** \| **Tree** \| **Deprecated** \| **Index** \| **Help** \| **Weka's home** | |  |
| **PREV CLASS**   **NEXT CLASS** | **FRAMES**    **NO FRAMES**     **All Classes** |  |
| SUMMARY: NESTED \| FIELD \| CONSTR \| METHOD | DETAIL: FIELD \| CONSTR \| METHOD |  |


---


## weka.gui.visualize Class VisualizePanelEvent

```
java.lang.Object
  weka.gui.visualize.VisualizePanelEvent
```

---

``` public class VisualizePanelEvent extends java.lang.Object ```

This event Is fired to a listeners 'userDataEvent' function when
The user on the VisualizePanel clicks submit. It contains the attributes
selected at the time and a FastVector containing the various shapes
that had been drawn into the panel.

**Version:**
:   $Revision: 1.4 $

**Author:**
:   Malcolm Ware (mfw4@cs.waikato.ac.nz)

---

| **Field Summary** | |
| --- | --- |
| `static int` | `HLINE` |
| `static int` | `LINE` |
| `static int` | `NONE`             No longer used |
| `static int` | `OVAL` |
| `static int` | `POLYGON` |
| `static int` | `RECTANGLE` |
| `static int` | `VLINE` |


**Constructor Summary**

`VisualizePanelEvent(FastVector ar,
Instances i,
Instances i2,
int at1,
int at2)`
  
          This constructor creates the event with all the parameters set.


| **Method Summary** | |
| --- | --- |
| `int` | `getAttribute1()` |
| `int` | `getAttribute2()` |
| `Instances` | `getInstances1()` |
| `Instances` | `getInstances2()` |
| `FastVector` | `getValues()` |

**Methods inherited from class java.lang.Object**

`equals, getClass, hashCode, notify, notifyAll, toString, wait, wait, wait`

**Field Detail**

### NONE

```
public static int NONE
```

:   No longer used

---


### RECTANGLE

```
public static int RECTANGLE
```

---


### OVAL

```
public static int OVAL
```

---


### POLYGON

```
public static int POLYGON
```

---


### LINE

```
public static int LINE
```

---


### VLINE

```
public static int VLINE
```

---


### HLINE

```
public static int HLINE
```


**Constructor Detail**

### VisualizePanelEvent

```
public VisualizePanelEvent(FastVector ar,
                           Instances i,
                           Instances i2,
                           int at1,
                           int at2)
```

:   This constructor creates the event with all the parameters set.

    **Parameters:**: `ar` - The list of shapes.: `i` - The instances that lie in these shapes.: `i2` - The instances that lie outside these shapes.: `at1` - The attribute that was along the x axis.: `at2` - The attribute that was along the y axis.


**Method Detail**

### getValues

```
public FastVector getValues()
```

:   **Returns:**: The list of shapes.

---


### getInstances1

```
public Instances getInstances1()
```

:   **Returns:**: The instances that lie in the shapes.

---


### getInstances2

```
public Instances getInstances2()
```

:   **Returns:**: The instances that lie outside the shapes.

---


### getAttribute1

```
public int getAttribute1()
```

:   **Returns:**: The x axis attribute.

---


### getAttribute2

```
public int getAttribute2()
```

:   **Returns:**: The y axis attribute.


---


|  |  |  |
| --- | --- | --- |
| **Overview** \| **Package** \| **Class** \| **Tree** \| **Deprecated** \| **Index** \| **Help** \| **Weka's home** | |  |
| **PREV CLASS**   **NEXT CLASS** | **FRAMES**    **NO FRAMES**     **All Classes** |  |
| SUMMARY: NESTED \| FIELD \| CONSTR \| METHOD | DETAIL: FIELD \| CONSTR \| METHOD |  |


---
